# Supplementary material for: The receptor for advanced glycation endproducts (RAGE) modulates T cell signaling
Source: PLoS One. 2020 Sep 28;15(9):e0236921. doi: 10.1371/journal.pone.0236921 (PMC7521722; doi:10.1371/journal.pone.0236921)
Supplement: S1 Fig — The primer pairs used for RAGE knockout detection were forward 5’-TGTTCCCCAGCCTTGCCTTCAT-3’ and reverse 5’-GCCCCTCCTCGCCTGGTTCT-3’, which generate a 533 bp PCR product unique to the RAGE gene deletion. Primers that were specific for the WT or excised fragment were prepared and the genomic DNA was analyzed by PCR. The absence of RAGE expression was confirmed by DNA amplification and FACS. (PDF) [file pone.0236921.s001.pdf]

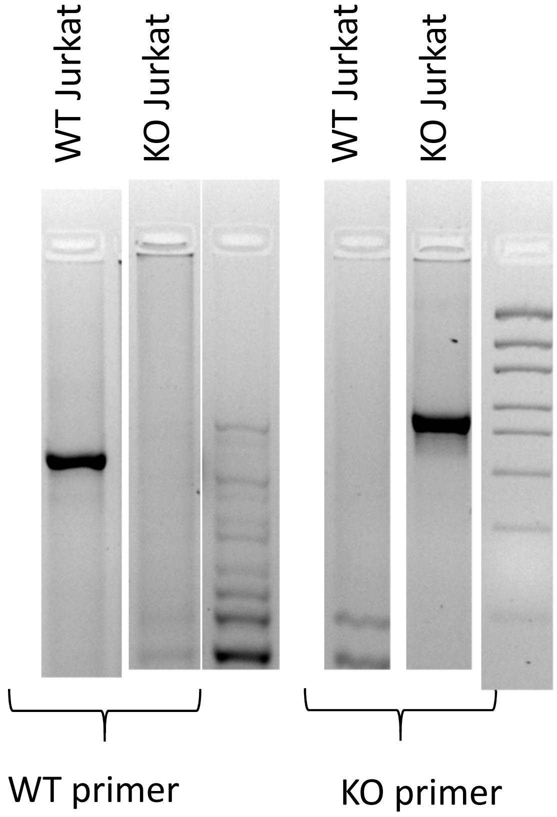

S1 Figure: Analysis of RAGE/KO Jurkat cells. The primer pairs used for RAGE knockout detection were forward 5'-TGTTCCCCAGCCTTGCCTTCAT-3' and reverse 5'-GCCCCTCCTCGCCTGGTTCT-3', which generate a 533 bp PCR product unique to the RAGE gene deletion. Primers that were specific for the WT or excised fragment were prepared and the genomic DNA was analyzed by PCR. The absence of RAGE expression was confirmed by DNA amplification and FACS.
