# Supplementary material for: The receptor for advanced glycation endproducts (RAGE) modulates T cell signaling
Source: PLoS One. 2020 Sep 28;15(9):e0236921. doi: 10.1371/journal.pone.0236921 (PMC7521722; doi:10.1371/journal.pone.0236921)
Supplement: S2 Fig — a: the frequency of Jurkat WT and KO cells that are positive for CD3 are shown (Student’s t-test, ***p<0.001,n = 8,6) b: The CD3 mean fluorescence index is shown (Student’s t-test, *p<0.05, n = 11,7), c: an example of staining. The filled bar shows the fluorescence of isotype control, the solid line shows the flurorescence of CD3 on the KO and the dotted line on the WT cells. (PDF) [file pone.0236921.s002.pdf]

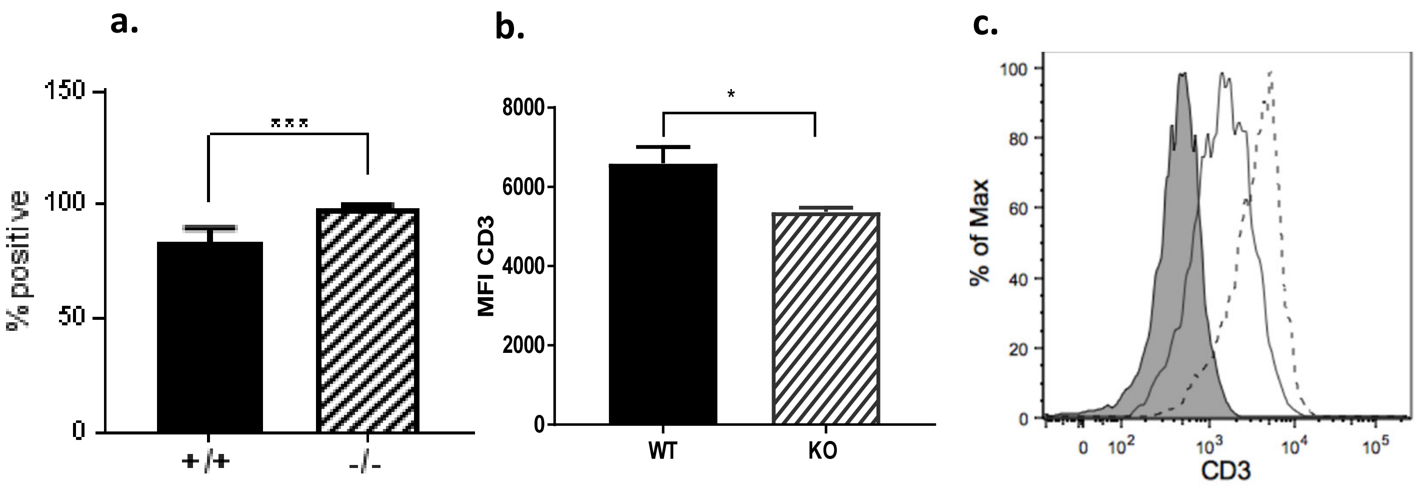

S2 Figure: CD3 expression in WT and RAGE KO Jurkat cells. a: the frequency of Jurkat WT and KO cells that are positive for CD3 are shown (Student's t-test, ***p<0.001,n=8,6) b: The CD3 mean fluorescence index is shown (Student's t-test, *p<0.05, n=11,7), c: an example of staining. The filled bar shows the fluorescence of isotype control, the solid line shows the flurorescence of CD3 on the KO and the dotted line on the WT cells.
