# Supplementary figures and images for: The receptor for advanced glycation endproducts (RAGE) modulates T cell signaling
Source: PLoS One. 2020 Sep 28;15(9):e0236921. doi: 10.1371/journal.pone.0236921 (PMC7521722; doi:10.1371/journal.pone.0236921)

# RAGE Gel

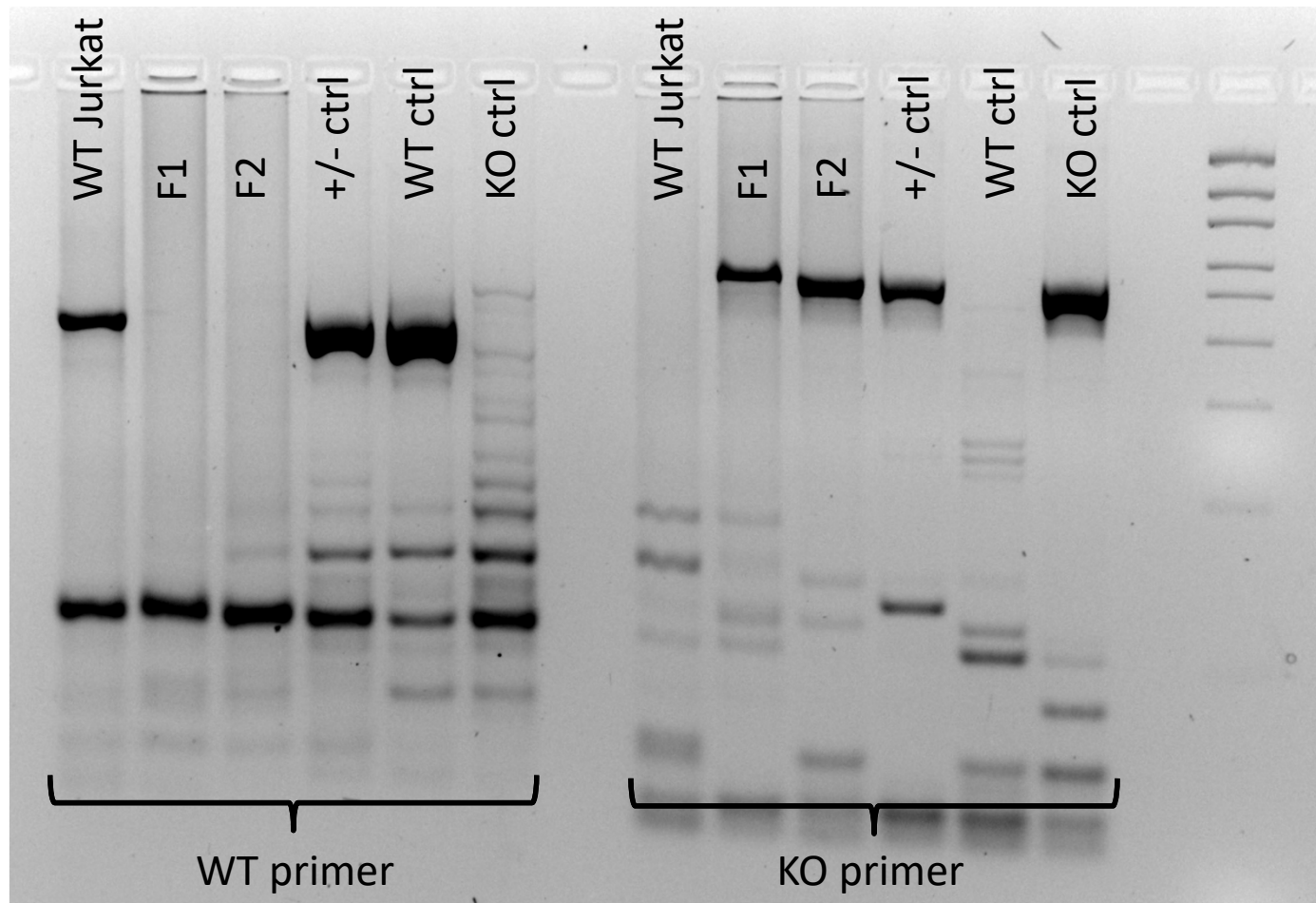

Supplement: S1 Raw image — (PDF) [file pone.0236921.s003.pdf]
