# Supplementary material for: The receptor for advanced glycation endproducts (RAGE) modulates T cell signaling
Source: PLoS One. 2020 Sep 28;15(9):e0236921. doi: 10.1371/journal.pone.0236921 (PMC7521722; doi:10.1371/journal.pone.0236921)
Supplement: Attachment — Submitted filename: plosresp.docx [file pone.0236921.s005.docx]

**Responses to review PONE-D-20-15463**

**Response to Associate Editor**

We agree with the importance of having a sufficient number of subjects to avoid type 1 and type 2 errors. We note that our previous version of the manuscript had an error in the numbers of patients analyzed – the correct numbers are 8 patients (not 3) with T1D and 6 healthy control subjects. The data from these patients and controls are shown in Figure 1.

We felt that it was important to give additional background on the studies that led to these investigations in T cell signaling as we have previously published information from 30 patients with T1D (including 7 in our PlosOne paper (Akirav et al PLoS One. 2012;7(4):e34698) and 23 patients at risk for T1D. The latter group, which involved participants in a TrialNet study, 9 progressed to overt T1D and 13 did not. We reported increased expression of RAGE in T cells prior to onset of T1D. Because of the extensive number of previously reported cases and characterization of the RAGE+ T cells, we did not feel that showing the frequency of RAGE in T cells from patients with T1D was again needed.

***Journal Requirements***

2. We have clarified the Jurkat cells that were used.
3. We have revised the Methods section as requested.
4. We have provided the uncropped gel that was used to generate Supplementary figure 1
5. We have removed the phrase “data not shown”. It refers to the transfection of the Jurkat cells with the commercially available RAGE siRNAs. It is a technical matter and we feel that that text description would be sufficient.

***Reviewer 1***

1. *Thank you for submitting your manuscript. In its current form, I can't support*

*publication. The data is limited to primary samples from 3 donors with T1D & 3 donors with*

*no history of disease along with cell line-based experiments.*

Response: Please see our response to the Editor. The manuscript includes data on 8 patients and 6 healthy control subjects. (Now with additional information about the patients.) This is in addition to the 52 other patients or at-risk subjects whom we have studied. In addition, in our previous publication (Durning et al J Immunol 2016), we described the phenotype of the RAGE+ CD4+ and CD8+ cells. They are memory cells that also express EOMES, KLRG1, IRF4, and CXCR3.

In addition to the above characterization, we have previously studied RAGE expression on viral antigen reactive cells (Akirav PlosOne 2012) and found expression on the antigen reactive cells (identified by Class I MHC tetramers) with increased expression when they were cultured with antigen.

We have added new text to the Background to review this information.

*2. Have you or do you plan to use the siRNA with PBMC that was used with the Jurkat?*

Response: For technical reasons, associated with silencing with siRNA in primary human T cells we chose instead to differentiate responses in RAGE+ and RAGE- human T cells, which is shown in Figure 1. The technical issues not only involve the difficulties in silencing in primary cells but also the need to activate and expand the primary cells prior to transfection with the siRNA. The activation and expansion itself enhances RAGE expression. Jurkat cells are frequently used for studies of T cell signaling. We felt, therefore, that studies in this cell line were relevant to human cells and our data supports that the lessons from the cell line are applicable.

*3. Other minor issues to be addressed:*

** Minimal donor information should be provided -- age & sex, at least?*

Response: thank you for this suggestion. We have now included this.

*4.	* Line 89, 95, 96, etc. – lower case ‘u’ is used in place of mu symbol—please correct all*

*instances throughout the manuscript*

Response: we corrected this error. Thanks for pointing it out.

5. ** Sentence on line#91-92 is poorly written & should be modified.*

Response: We agree and changed the wording.

6. ** Tenses are inconsistent throughout the manuscript. In general, methods sections should be past tense.*

Response: We have reworded the methods section and clarified a number of the sentences.

7. ** Line 217 – space between ‘without’ and ‘anti-CD28’*

Response: we corrected this error.
