# Supplementary material for: The receptor for advanced glycation endproducts (RAGE) modulates T cell signaling
Source: PLoS One. 2020 Sep 28;15(9):e0236921. doi: 10.1371/journal.pone.0236921 (PMC7521722; doi:10.1371/journal.pone.0236921)
Supplement: Attachment — Submitted filename: plos3resp.docx [file pone.0236921.s006.docx]

**PONE-D-20-15463R1**

Thank you for the review of our manuscript and your email of 7/20/3030. We were please to learn that the Reviewers’ felt that we had responded to their queries. Below please find a point-by-point response to the remaining queries (the queries are shown in Italics):

1) *For the methods, a brief description of the cryopreservation should be added.*

We have added text under the subheading “Cell lines and human samples”

*2) line 89-90 -- authors state that they have 8 participants with T1D but the demographic description says 2 M & 5 F, they are missing 1 participant in their count. Additionally, no information regarding the healthy subjects--were they similar in sex distribution & age?*

Thank you for pointing out this error. We have fixed the text. There are 8 patients (6F, 2M). Figure 1 includes the data on all 8. We obtained buffy coats from the New York Blood Center. These donors are de-identified and the only information we are given is that they were adults and did not have significant medical problems.

*3) Were the PBMC rested for any time post-thaw before culture & stimulation?*

No, they were not. We clarified the methods to indicate exactly how the experiments were done.

*4) line 166 -- author states they studied T-cells from healthy & donors with T1D and it was n = 3 each -- why weren't all donors evaluation? Or is this a typo? The figure description list n = 8 & 6 for T1D & healthy, respectively.*

We apologize for the error. It was a typo. The correct numbers are in the Figure legend: n=8 for patients with T1D and n=6 Healthy control subjects.
